# NopC Is a *Rhizobium*-Specific Type 3 Secretion System Effector Secreted by *Sinorhizobium* (*Ensifer*) *fredii* HH103

**DOI:** 10.1371/journal.pone.0142866

**Published:** 2015-11-16

**Authors:** Irene Jiménez-Guerrero, Francisco Pérez-Montaño, Carlos Medina, Francisco Javier Ollero, Francisco Javier López-Baena

**Affiliations:** 1 Departamento de Microbiología, Facultad de Biología, Universidad de Sevilla, Sevilla, Spain; 2 Centro Andaluz de Biología del Desarrollo, Universidad Pablo de Olavide, Consejo Superior de Investigaciones Científicas, Junta de Andalucía, Sevilla, Spain; Centre National de la Recherche, FRANCE

## Abstract

*Sinorhizobium* (*Ensifer*) *fredii* HH103 is a broad host-range nitrogen-fixing bacterium able to nodulate many legumes, including soybean. In several rhizobia, root nodulation is influenced by proteins secreted through the type 3 secretion system (T3SS). This specialized secretion apparatus is a common virulence mechanism of many plant and animal pathogenic bacteria that delivers proteins, called effectors, directly into the eukaryotic host cells where they interfere with signal transduction pathways and promote infection by suppressing host defenses. In rhizobia, secreted proteins, called nodulation outer proteins (Nops), are involved in host-range determination and symbiotic efficiency. *S*. *fredii* HH103 secretes at least eight Nops through the T3SS. Interestingly, there are *Rhizobium*-specific Nops, such as NopC, which do not have homologues in pathogenic bacteria. In this work we studied the *S*. *fredii* HH103 *nopC* gene and confirmed that its expression was regulated in a flavonoid-, NodD1- and TtsI-dependent manner. Besides, *in vivo* bioluminescent studies indicated that the *S*. *fredii* HH103 T3SS was expressed in young soybean nodules and adenylate cyclase assays confirmed that NopC was delivered directly into soybean root cells by means of the T3SS machinery. Finally, nodulation assays showed that NopC exerted a positive effect on symbiosis with *Glycine max* cv. Williams 82 and *Vigna unguiculata*. All these results indicate that NopC can be considered a *Rhizobium*-specific effector secreted by *S*. *fredii* HH103.

## Introduction

Rhizobia are soil bacteria able to establish a symbiotic interaction with legumes that culminates in the formation of specialized plant organs, called nodules, on the roots of the host plant. Within these symbiotic structures atmospheric nitrogen is reduced to ammonia, which is assimilated by the host plant in exchange of a carbon source and an appropriate environment that promotes bacterial growth [1]. This process requires a complex interchange of molecular signals between the microorganism and the plant. Thus, certain flavonoids exuded by legume roots are recognized by the rhizobial protein NodD, which in turns binds to specific promoter sequences (*nod* boxes), activating the transcription of the *nod* genes. Proteins encoded by these genes are responsible for the biosynthesis and secretion of the Nod factors, which are recognized by specific plant receptors to initiate nodule organogenesis [2].

Plant flavonoids, besides inducing Nod factors production, attract the bacteria to the legume root [3], activate the rhizobial quorum sensing systems [4,5], and induce via NodD the secretion of proteins through the type 3 secretion system (T3SS) [6]. This specialized secretion apparatus is a common virulence mechanism shared by many plant and animal pathogenic Gram negative bacteria that delivers proteins directly into the host cells [7,8,9]. These secreted proteins are called effectors and function within the eukaryotic cell, where they interfere with signal transduction cascades and promote infection by suppressing host defenses [10,11].

In rhizobia, secreted proteins are collectively known as nodulation outer proteins (Nops) [12] and are involved in host-range determination and symbiotic efficiency [13]. Recent works have shown that the *S*. *fredii* HH103 T3SS is responsible of the suppression of early soybean defense responses to effectively nodulate this legume [14]. In addition, the T3SS of *Bradyrhizobium elkanii* USDA61 induces the formation of nodules in soybean in the absence of Nod factors when infecting by crack-entry or intercellular infection [15]. Synthesis and secretion of Nops are controlled by the transcriptional regulator TtsI, which binds to specific promoter sequences called *tts* boxes. TtsI is an intermediary in the regulatory cascade between NodD, previously activated by flavonoids, and the T3SS-related genes [6,16,17,18].

*S*. *fredii* HH103, hereafter HH103, is a broad host-range bacterium that nodulates many legumes including soybean, which is considered its natural host plant [19]. HH103 secretes at least eight proteins through the T3SS in response to the inducer flavonoid genistein: NopA, NopB, NopC, NopD, NopL, NopM, NopP, and NopX [20]. NopA, NopB, and NopX are extracellular components of the T3SS machinery [21,22,23] and the rest can be considered putative effectors (NopC, NopD, NopL, and NopM) with the exception of NopP, whose secretion to the interior of *Vigna unguiculata* nodule cells has been confirmed [24]. Interestingly, two of these proteins, NopL and NopP, are specific to rhizobia and have no homologues in plant or animal pathogens [13]. NopL is phosphorylated by plant kinases and probably interferes with plant signal transduction cascades that are responsible of the activation of plant defense genes [25]. In addition, NopL seems to be involved in the suppression of the nodule premature senescence observed in the symbiosis between *S*. *fredii* NGR234 and *Phaseolus vulgaris* [26]. NopP has also been described as phosphorylated by plant kinases but its function in symbiosis is still unknown [27]. In HH103, the inactivation of the *nopP* gene causes an increase in the number of nodules formed in American and Asiatic soybeans [28]. Finally, no reports about the possible function or the role in symbiosis of NopC have been published so far.

In this work, we studied the transcriptional regulation of the *nopC* gene by NodD1, TtsI and flavonoids and the effect of a non-polar mutation in *nopC* on Nops secretion. We determined that the *tts* region of HH103 was expressed in young soybean nodules and showed that NopC was translocated into soybean root cells, confirming that this protein can be considered an effector secreted by the HH103 T3SS. Finally, we studied the role of NopC in the symbiosis with *Glycine max* (soybean) cv. Williams 82 and *V*. *unguiculata*.

## Materials and Methods

### Microbial and molecular techniques

Bacterial strains and plasmids used in this work are listed in Table 1. *Sinorhizobium* strains were grown at 28°C on tryptone yeast (TY) medium [29] or yeast extract mannitol (YM) medium [30]. *Escherichia coli* strains were cultured on Luria-Bertani (LB) medium [31] at 37°C. When required, the media were supplemented with the antibiotics ampicillin (Ap, 100 μg ml^-1^), rifampicine (Rif, 50 μg ml^-1^), spectinomycin (Spc, 50 μg ml^-1^), kanamycine (Km, 30 μg ml^-1^), tetracycline (Tc, 5 μg ml^-1^), and gentamycine (Gm, 5 μg ml^-1^). Genistein was dissolved in ethanol and used at 1 μg ml^-1^ to give a final concentration of 3.7 μM. Plasmids were transferred from *E*. *coli* to *Sinorhizobium* strains by conjugation as described by [32] using plasmid pRK2013 as helper.

**Table 1:** Bacterial strains and plasmids.

Strain or plasmid	Relevant properties	Source or reference
*Sinorhizobium fredii* HH103		
HH103 Rif^R^	Parental strain; Rif^R^	[46]
HH103 Rif^R^ (pMUS1199)	HH103 Rif^R^ carrying plasmid pMUS1199; Tc^R^	This work
HH103 Rif^R^ (pMUS1207)	HH103 Rif^R^ carrying plasmid pMUS1207; Tc^R^	This work
HH103 Rif^R^ *nodD1*::*lacZ*-Gm^R^	HH103 Rif^R^ mutant derivative with the *lacZ*-Gm^R^ cassette inserted into *nodD1*; Gm^R^	[47]
HH103 Rif^R^ *nodD1*::*lacZ*-Gm^R^(pMUS1207)	*nodD1* mutant carrying plasmid pMUS1207; Gm^R^ Tc^R^	This work
HH103 Rif^R^ *ttsI*::Ω	HH103 Rif^R^ mutant derivative with the Ω interposon inserted into *ttsI*; Spc^R^	[17]
HH103 Rif^R^ *ttsI*::Ω (pMUS1207)	*ttsI* mutant carrying plasmid pMUS1207; Spc^R^ Tc^R^	This work
HH103 Rif^R^ *nopA*::*lacZ*-Gm^R^	HH103 Rif^R^ mutant derivative with the *lacZ*-Gm^R^ cassette inserted into *nopA*; Gm^R^	[17]
HH103 Rif^R^ Δ*nopC*	HH103 Rif^R^ mutant derivative with a deletion of the *nopC* gene	This work
HH103 Rif^R^ Δ*nopC* complemented in *cis*	*nopC* mutant complemented in *cis* by conjugation and simple recombination of plasmid pMUS1192, Km^R^	This work
HH103 Rif^R^ Δ*nopC* (pMUS986)	*nopC* mutant complemented with plasmid pMUS986; Tc^R^	This work
HH103 Rif^R^ *nopC*::*cya*	HH103 Rif^R^ with the *nopC*-*cya* fusion integrated in the chromosome; Km^R^	This work
HH103 Rif^R^ *ttsI*::Ω *nopC*::*cya*	*ttsI* mutant with the *nopC*-*cya* fusion integrated in the chromosome, Km^R^	This work
*Escherichia coli*		
DH5α	*supE44*, Δ*lacU169*, *hsdR17*, *recA1*, *endA1*, *gyrA96*, *thi-1*, *relA1*; Nx^R^	[31]
DB3.1	*F-*, *gyrA462*, *endA1*, *glnV44*, Δ*(sr1-recA)*, *mcrB*, *mrr*, *hsdS20 (r*_*B*_^*-*^, *m*_*B*_^*-*^ *)*, *ara14*, *galK2*, *lacY1*, *proA2*, *rpsL20* (Sm^r^), *xyl5*, Δ*leu*, *mtl1*; Nx^R^	Invitrogen
Plasmids		
pGEM-T Easy	PCR cloning vector; Ap^R^	Promega
pAB2001	Vector containing the *lacZ*-Gm^R^ cassette; Ap^R^	[48]
pK18*mob*	Cloning vector, suicide in rhizobia; Km^R^	[35]
pK18*mobsac*	Cloning vector, suicide in rhizobia; Km^R^	[35]
pMP92	Broad host-range cloning vector, IncP; Tc^R^	[33]
pRK2013	Helper plasmid; Km^R^	[49]
Flux vector	Plasmid miniCTX1 containing the *luxCDABE* operon	Provided by Dr. Cámara
pDONR207	Entry vector in Gateway technology; Gm^R^	Invitrogen
pLMS150	Destination vector in for *cya* gene fusion; Tc^R^	[24]
pMUS986	pMP92 carrying an HH103 1.3-kb DNA fragment containing *nopC*, *nopA* and their upstream *tts* box	[17]
pMUS1192	pK18*mobsac* carrying a 1.1-kb DNA fragment containing Δ*nopC*; Km^R^	This work
pMUS1199	pMP92 carrying the *luxCDABE* operon; Tc^R^	This work
pMUS1207	pMP92 carrying a *tts* box-*luxCDABE* fusion; Tc^R^	This work
pMUS1239	pDONR207 carrying the *nopC* gene; Gm^R^	This work
pMUS1290	pLMS150 carrying the *nopC* gene; Tc^R^	This work
pMUS1291	pK18*mob* carrying a 1.5-kb fragment containing the *nopC-cya* fusion; Km^R^	This work

Recombinant DNA techniques were performed according to the general protocols of [31]. For hybridization, DNA was blotted to Hybond-N nylon membranes (Amersham, United Kingdom), and the DigDNA method of Roche (Switzerland) was employed following the manufacturer's instructions. PCR amplifications were performed as previously described [5]. Primer pairs used for the amplification of the *S*. *fredii* HH103 *nopC* and *nopA* genes were fy1secF and fy1secR (Table 2). Plasmid pMUS986 was obtained by cloning into the broad host-range vector pMP92 [33] a 1.3-kb PCR fragment containing the *nopC* and *nopA* genes and their upstream *tts* box.

**Table 2:** DNA oligonucleotide primers used in this study.

Name	Sequence	Usage
fy1sec F	5'-CCAGGGAGTCCAGATCGTGCA-3'	Amplification of *nopC* and *nopA*
fy1sec R	5'-GAGGCGTGGTTTACCGATCGA-3'	
nopC-1	5'-ATTAAGCTTTGTCATGGACAGGGAACGAA-3'	*nopC in frame* deletion
nopC-2	5'-CAGTTTCTGCCATACCACTTCCAATCAC-3'	
nopC-3	5'-GTGATTGGAAGTGGTATGGCAGAAACTG-3'	
nopC-4	5'-AAAGGATCCGCGAAATGGCGTCGTTCACT-3'	
attBnopC1	5'-GGGGACAAGTTTGTACAAAAAAGCAGGCTTAATGGTCGGAGTGATTGGA-3'	Gateway vector cloning
attBnopC2	5'-GGGGACCACTTTGTACAAGAAAGCTGGGTAGGCATCCTCTTCAGTTTC-3'	
nopC_EcoRI F	5'-AAAGAATTC **ATG**GTCGGAGTGATTGGAAG-3'	Amplification of the *nopC-cya* fusion
cya_BamHI R	5'-ATAGGATCC **TCA**GCTGTCATAGCCGGAAT-3'	
nopCq_F	5'-CAAAGGGGGGCATGGA-3'	*q*RT-PCR assays
nopCq_R	5'-CAACCGATCGAAGAGCTA-3'	
nopAq_F	5'-TGTCACGAGTGCAGTTGGA-3'	*q*RT-PCR assays
nopAq_R	5'-TGTCTGGAGCTCGGTCGTAA-3'	
nodAq_F	5'-CGTCATGTATCCGGTGCTGCA-3'	*q*RT-PCR assays
nodAq_R	5'-CGTTGGCGGCAGGTTGAGA-3'	
16Sq_F	5'-TAAACCACATGCTCCACC-3'	*q*RT-PCR assays
16Sq_R	5'-GATACCCTGGTAGTCCAC-3'	
ttsbox F	5'-AAAAAGCTTCAACTGCACACGTTAGCGTT-3'	*tts* box amplification
ttsbox R	5'-AAAGAATTCCAGCTACTCCTGCCTTAGCG-3'	

The *in frame nopC* gene deletion was constructed by overlap extension polymerase chain reaction [34] using the pairs of primers nopC-1/nopC-2 and nopC-3/nopC-4 (Table 2). Plasmid pK18*mobsac* was used for the homogenotization of the mutated version of *nopC* in *S*. *fredii* HH103 Rif^R^ [35]. The deletion event was confirmed by PCR and hybridization. The *nopC* deletion was complemented in *trans* by the transference of plasmid pMUS986 and in *cis* by single recombination using plasmid pMUS1192.

Construction of the translational fusion *nopC-cya* was performed by cloning a DNA fragment containing the *nopC* gene without end codon, which was amplified using primers listed in Table 2, into plasmid pDONR207 (Invitrogen, USA). The resulting plasmid (pMUS1239) was used to clone the *nopC* gene into plasmid pLMS150 [24], which possesses recombination sites for the clonase II upstream the *cya* gene, resulting in plasmid pMUS1290. This gene fusion was confirmed by sequencing. Two primers (Table 2) were designed to amplify the *nopC-cya* fusion from pMUS1290 and add *Eco*RI and *Bam*HI restriction sites. Finally, the 1.5-kb PCR product was digested with *Eco*RI and *Bam*HI and the resulting DNA fragment was cloned into pK18 *mob* to obtain plasmid pMUS1291. This plasmid was used for the integration of the *nopC-cya* fusion by single recombination in the symbiotic plasmids of *S*. *fredii* HH103 Rif^R^ and in its *ttsI*Ω mutant derivative. These integrations were confirmed by PCR amplification using primers described previously (Table 2). Secretion of the NopC-Cya fusion protein by the T3SS was confirmed using specific antibodies against the cya epitope (data not shown).

### RNA isolation, cDNA synthesis and quantitative RT-PCR

*q*RT-PCR assays were performed as described by [4]. Briefly, *S*. *fredii* strains HH103 Rif^R^, HH103 Rif^R^ *nodD1*::*lacZ*-Gm^R^, HH103 Rif^R^ *ttsI*::*Ω*, and HH103 Rif^R^Δ*nopC* were grown with shaking at 28°C in YM medium supplemented with genistein when necessary. When cultures reached an OD_600_ of 0.8, cells were harvested and RNA was extracted using the High Pure RNA Isolation kit following the manufacturer’s instructions (Roche, Switzerland). Two independent RNA extractions were performed. RNA concentration was quantified using a Nanodrop 2000 spectrophotometer (Thermo Scientific, USA) and the integrity of the RNA was assayed on 2% agarose gels. cDNA was synthesized using the Quantitect Reverse Transcription kit according to the manufacturer’s instructions (Qiagen, Switzerland).

To quantify the HH103 *nopC*, *nopA*, and *nodA* gene expression using quantitative RT-PCR, primers nopCq_F, nopCq_R, nopAq_F, nopAq_R, nodAq_F, and nodAq_R were designed (Table 2). The reactions were performed in a 10 μl final volume containing 25 ng of cDNA, 0.6 pmol of each primer and 5 μl of FastStart SYBR Green Master Mix (Roche, Switzerland). PCR was conducted on a Light Cycler 480 II (Roche) with the following conditions: 95°C, 10 min; 95°C, 30 sec; 50°C, 30 sec; 72°C, 15 sec; 45 cycles, followed by the melting curve profile from 65 to 95°C to verify the specificity of the reaction. The threshold cycles (Ct) were determined with the Light Cycler 480 II software and the individual values for each sample were generated by averaging three technical replicates that varied less than 0.5 cycles. Expression was calculated relative to the parental strain grown without flavonoids. The HH103 RNA *16S* gene was used as an internal control to normalize gene expression (Table 2). The fold change in the target gene, normalized to RNA *16S* and relative to the gene expression in the control sample was calculated.

### Purification and analysis of nodulation outer proteins

Extracellular proteins from several HH103 strains were recovered from 50 ml of YM bacterial cultures grown on an orbital shaker (180 r.p.m.) for 40 h (approximately 10^9^ c.f.u. ml^-1^). Cultures were centrifuged for 20 min at 10000 *g* at 4°C. The supernatants were mixed with 3 volumes of cold acetone and maintained at -20°C for 24 h. The mixtures were centrifuged for 45 min at 22000 *g* at 4°C. Dried pellets were resuspended in 300 μl of sample buffer (62.5 mM Tris-HCl [pH 6.8], 2% SDS [m/v], 10% glycerol [v/v], 5% β-mercaptoethanol [m/v], and 0.001% bromophenol blue [m/v]). Extracellular proteins were separated by SDS-PAGE using the discontinuous buffer system of Laemmli [36]. Electrophoresis was performed on SDS 15% (m/v) polyacrylamide gels and proteins were visualized by silver staining.

For immunostaining, extracellular proteins were separated on SDS 15% (m/v) polyacrylamide gels and electroblotted to Immun-Blot polyvinylidene difluoride membranes (Bio-Rad, USA) using a Mini Trans-Blot electrophoretic transfer cell (Bio-Rad). Membranes were blocked with TBS containing 2% (m/v) bovine serum albumin (BSA) and then incubated with antibodies raised against NopA, NopB, NopC, NopP, and NopX [21] or the cya epitope (Cell Signaling Technologies, USA) diluted 1:1000 in the same solution. Anti-rabbit immunoglobulin AP-conjugated secondary antibody was used and reaction results were visualized using NBT-BCIP.

### *In vivo* monitoring of the activation of the *tts* box upstream *nopC* during the initial stages of the symbiosis with soybean

The Flux vector (Table 1) was digested with *Bam*HI and *Eco*RI and the resulting 5.8 kb DNA fragment carrying the *luxCDABE* genes was cloned into the broad host-range plasmid pMP92 to obtain plasmid pMUS1199. Two primers (Table 2) were designed to amplify the *tts* box upstream *nopC* from pMUS986 and add *Eco*RI and *Hin*dIII restriction sites. The resulting ~0.2 kb PCR fragment was digested with the restriction enzymes indicated previously and cloned into plasmid pMUS1199 previously digested with *Eco*RI and *Hin*dIII, obtaining plasmid pMUS1207. The *luxCDABE* operon encodes the luciferase enzyme and therefore instant *in vivo* promoter activity can be monitored by measuring bioluminescence. Then, two different experiments were carried out. In the first one, nine pre-germinated *G*. *max* cv. Williams 82 seeds were aseptically transferred to a recipient containing vermiculite and 150 ml of a Fåhraeus solution 1M pH 6.8 and were grown in a controlled environment chamber with a 16 h day/8 h night cycle and a relative humidity of 70%. Growth temperatures were set to 26°C during the day period and to 18°C during the night. The system was inoculated at the time of transferring the pre-germinated seeds with a bacterial culture of about 10^8^ c.f.u. ml^-1^ of *S*. *fredii* HH103 Rif^R^ carrying plasmid pMUS1207 or plasmid pMUS1199. In the second one, pre-germinated seeds were placed on sterilized pouches containing the same nitrogen-free Fåhraeus solution, inoculated with 1 ml of the previously described bacterial strains and the formation of nodules and bioluminescence were monitored at 14, 16, 19, 21, 23, 26, and 29 days post-inoculation (d.p.i.). In both cases experiments were performed twice with five replicates and bioluminescence was quantified using a photon-counting camera IVIS Lumina II (Caliper Life Science, USA). Images were analyzed with the Living Image 4.0 software (Caliper Life Science). A root-flavonoid diffusion assay was used to validate the T3SS-dependent bioluminescence. Thus, pre-germinated soybean seeds were placed in squared Petri dishes and roots were covered with a volume of 20 ml of TY (1.2% agar) mixed with 10 ml bacterial cultures of strains HH103 Rif^R^ (pMUS1207), HH103 Rif^R^ *nodD1*::*lacZ*-Gm^R^ (pMUS1207), HH103 Rif^R^ *ttsI*::Ω (pMUS1207), and HH103 Rif^R^ (pMUS1199). Bioluminescence was quantified 72 h after inoculation.

### Adenylate cyclase (cya) assay

To examine whether *S*. *fredii* HH103 T3SS translocates the NopC-Cya fusion protein into soybean root cells, the protocol described by [24] was used with some modifications. Eighteen pre-germinated soybean seeds were aseptically transferred to a recipient containing vermiculite and 150 ml of a Fåhraeus solution 1M pH 6.8 and grown in a controlled environment chamber with a 16 h day/8 h night cycle and a relative humidity of 70%. Growth temperatures were set to 26°C during the day period and to 18°C during the night. The system was inoculated at the time of transferring the pre-germinated seeds with bacterial cultures of about 10^8^ c.f.u.ml^-1^ of the HH103 Rif^R^ or the *ttsI*Ω mutant strains, both containing a chromosomal integration of the *nopC-cya* fusion. Cyclic AMP (cAMP) accumulation was measured in nodules harvested 18 d.p.i. Nodules were frozen in liquid nitrogen, ground to a fine powder and resuspended in a 0.1 M hydrochloric acid solution. The suspension was centrifuged and the supernatant was used for cAMP measurement using the cyclic AMP (direct) EIA kit (Cayman Chemical Company, USA) according to the manufacturer’s instructions. Each sample was diluted for quantification to measure cAMP concentration in the detection range of the assay. The HH103 Rif^R^ strain without the *nopC*-*cya* fusion was used as a control for quantification.

### Plant assays

Nodulation assays on *G*. *max* cv. Williams 82 or *V*. *unguiculata* were performed as described by [37]. Each Leonard jar contained two soybean or two *V*. *unguiculata* plants. Each plant was inoculated with about 10^8^ c.f.u. ml^-1^. Plants were grown for about 42 days with a 16 hour-photoperiod at 25°C in the light and 18°C in the dark. Plant tops were dried at 70°C for 48 h and weighed.

## Results

### NopC is a *Rhizobium*-specific T3SS secreted protein

**Fig 1:**
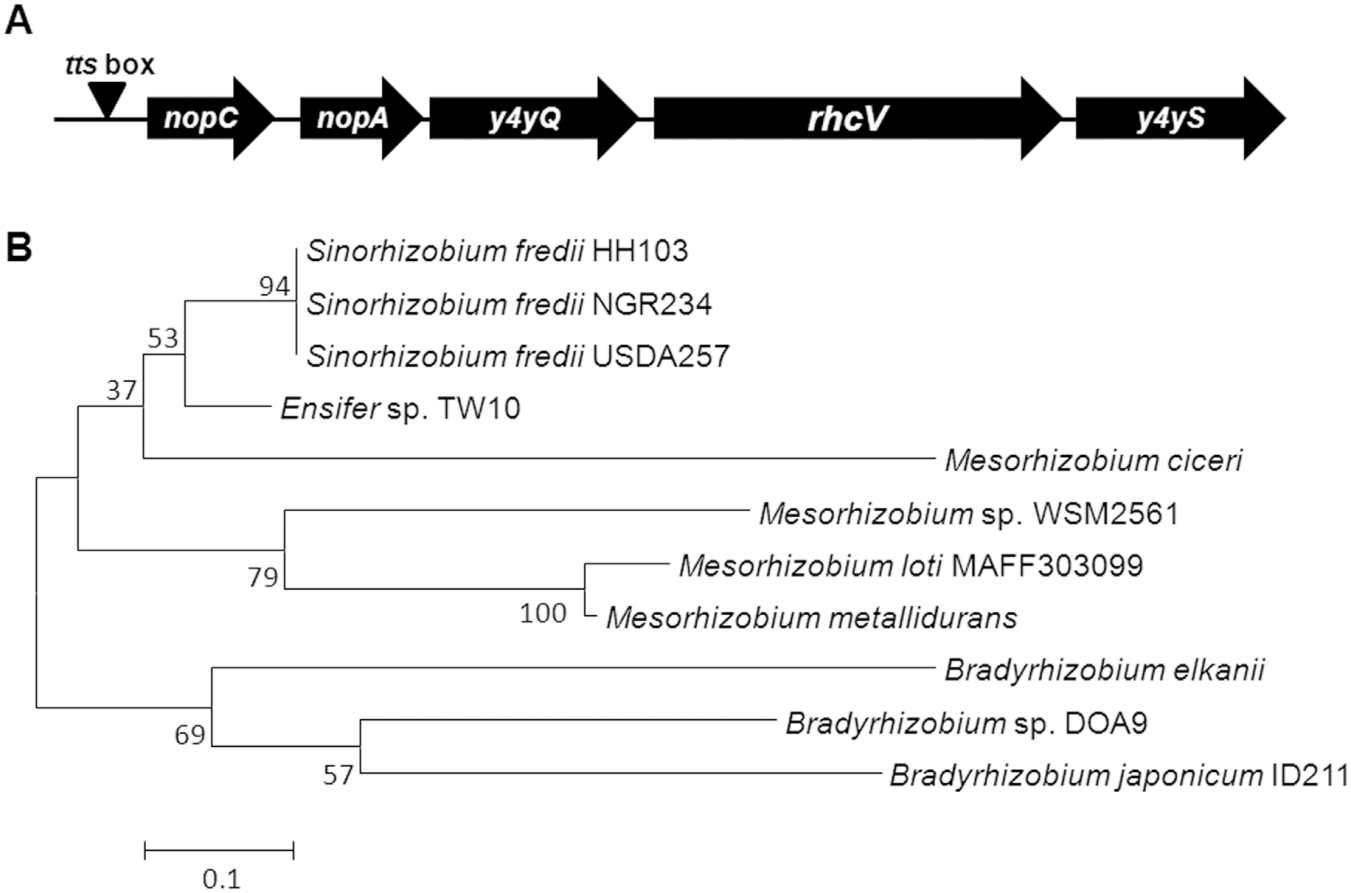
Organization of the HH103 *nopC* locus. (A) Position of the annotated open reading frames (ORFs) *nopC*, *nopA*, *y4yQ*, *rhcV*, and *y4yS*. (B) Neighbor joining phylogenies of the NopC tree of several rhizobial strains. Bootstrap values ≥ 60 are indicated for each node. The cluster analysis to group the strains by NopC sequence similarity was done using the program CLUSTAL W in the MEGA5 software package with the algorithm neighbor-joining method. Tree robustness was assessed by bootstrap resampling (1000 replicates each).

The *nopC* and *nopA* genes are located in the symbiotic plasmid of HH103 (pSfHH103d). The analysis of the genome sequence of HH103 revealed the presence of a conserved *tts* box situated 43 pb upstream *nopC* (297 pb). The *nopA* gene (216 pb) was located 80 pb downstream *nopC* (Fig 1a). Therefore, the organization and position of *nopC* within the *tts* region was similar to those described in *S*. *fredii* NG234 [21].

The genome of HH103 contains two genes, *nopL* and *nopP*, which code for two type 3-secreted proteins exclusive of rhizobia. NopC also seems to be *Rhizobium*-specific since no homologues have been detected in animal or plant pathogens. Neighbor joining phylogenies of the NopC tree of several rhizobial strains showed two clearly separated branches. At the bottom of the branch we found the three *Bradyrhizobium* species in which the NopC protein has been identified and annotated. On the other hand, the top branch is split between *Mesorhizobium* and *Sinorhizobium* (*Ensifer*) species. Interestingly, the neighbor joining of the NopC sequence of the *S*. *fredii* strains showed identical gene sequence (Fig 1b). This result was confirmed by aligning the HH103 (AAY33494), USDA257 (not annotated in the databases) and NGR234 (YP_052972) NopC sequences and verifying 100% sequence identity (data not shown).

**Fig 2:**
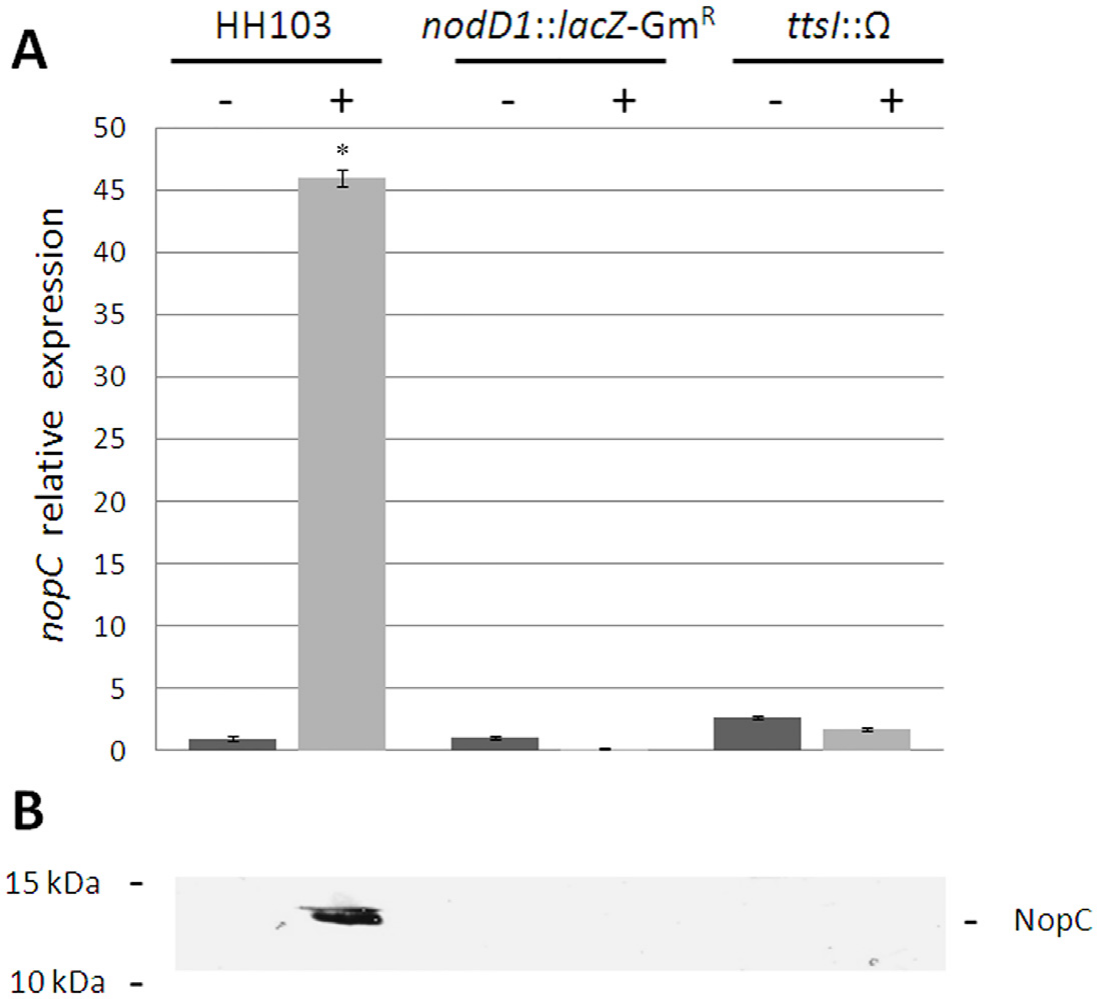
The expression of the *S*. *fredii* HH103 *nopC* gene is regulated by flavonoids, NodD1 and TtsI. (A) *q*RT-PCR analysis of the expression of *nopC* in the parental strain HH103 Rif^R^ and the mutant strains HH103 Rif^R^ *nodD1*::*lacZ*-Gm^R^ and HH103 Rif^R^ *ttsI*::Ω in the absence (-) or presence (+) of the inducer flavonoid genistein (3.7 μM). Final expression was calculated relative to the expression of the HH103 Rif^R^ strain in the absence of flavonoids. Expression data shown are the mean (± standard deviation of the mean) for two biological replicates performed at least in triplicates. Each expression value was individually compared with the HH103 Rif^R^ strain in the absence of flavonoids using the Mann-Whitney non-parametrical test. Asterisks indicate that numbers are significantly different at the level α = 5% (*p*< 0.05). (B) Immunodetection of NopC in extracellular proteins extracts of the parental strain HH103 Rif^R^ and the *ttsI* and *nodD1* mutants in the presence or absence of genistein (3.7 μM). Molecular masses (kDa) of the marker are shown on the left. Samples were separated by 15% SDS-PAGE.

Previous works have shown that the HH103 *nopA* expression is regulated in a flavonoid-, NodD1- and TtsI-dependent manner [17]. Therefore, it was presumable that the expression of the *nopC* gene was regulated in the same manner because both genes are preceded by the same *tts* box. This was confirmed by quantitative real-time PCR assays in which the expression of *nopC* in the parental strain HH103 Rif^R^ and in a *nodD1* and a *ttsI* mutant backgrounds, in the absence or presence of the flavonoid genistein, was studied. As shown in Fig 2a, induction with genistein increased the transcription of *nopC* about 45-fold in the parental strain. By contrast, transcription of this gene was not detected either in the *nodD1* or the *ttsI* mutants, confirming that the expression of *nopC* depends on flavonoids and on the transcriptional regulators NodD1 and TtsI.

Previous reports detected NopC in the supernatants of NGR234 and HH103 cultures grown in the presence of inducer flavonoids [20,21]. To confirm that secretion of NopC depends, together with flavonoids, on NodD and TtsI, proteins from strains HH103 Rif^R^, HH103 Rif^R^ *nodD1*::*lacZ*-Gm^R^, and HH103 Rif^R^ *ttsI*::Ω culture supernatants, in the presence or absence of inducing flavonoids, were extracted and separated by SDS-PAGE. The band of about 11 kDa corresponding to NopC was only observed in the supernatants of HH103 Rif^R^ cultures induced with genistein and not in the *nodD1* and *ttsI* mutants (data not shown). As expected, western-blots assays showed that the antibody raised against NopC specifically detected a protein of about 11 kDa in these supernatants. No signal was detected in the lanes corresponding to the *nodD1* or *ttsI* mutant strains with or without genistein (Fig 2b).

### *In frame* deletion of *nopC* did not alter secretion of other nodulation outer proteins or the expression of *nopA*

As previously mentioned, *nopA* was located downstream *nopC* in the HH103 genome. Previous reports indicated that the inactivation of the NGR234 *nopA* gene completely abolished secretion of Nops and confirmed, together with the analysis of the interaction of NopA with NopB and NopX, that NopA was the major component of the T3SS *pilus* [21]. In HH103, the mutation of *nopA* also abolished secretion of Nops to the extracellular medium (data not shown). As the *nopC* and *nopA* genes are transcribed from the same *tts* box, the construction of an *in frame* mutation of the *nopC* gene was necessary to study the effect of the inactivation of *nopC* on protein secretion because a polar mutation would block secretion due to its effect on *nopA* transcription. Therefore, to confirm that the expression of *nopA* was not affected by the mutation, transcription of *nopA* in the Δ*nopC* mutant background was quantified by quantitative real-time PCR in strains HH103 Rif^R^, HH103 Rif^R^ Δ*nopC*, and HH103 Rif^R^ *ttsI*::*Ω* in the absence or presence of the flavonoid genistein. As shown in S1 Fig, induction with genistein increased the transcription of *nopA* about 34-fold and 32-fold in the parental and the *nopC* mutant strain, respectively. This increase in gene expression was not detected in the *ttsI* mutant induced with flavonoids. As a control, expression of *nodA* was quantified in all the treatments previously analyzed. Results showed increased expression in the presence of genistein in all the strains assayed (S1 Fig).

**Fig 3:**
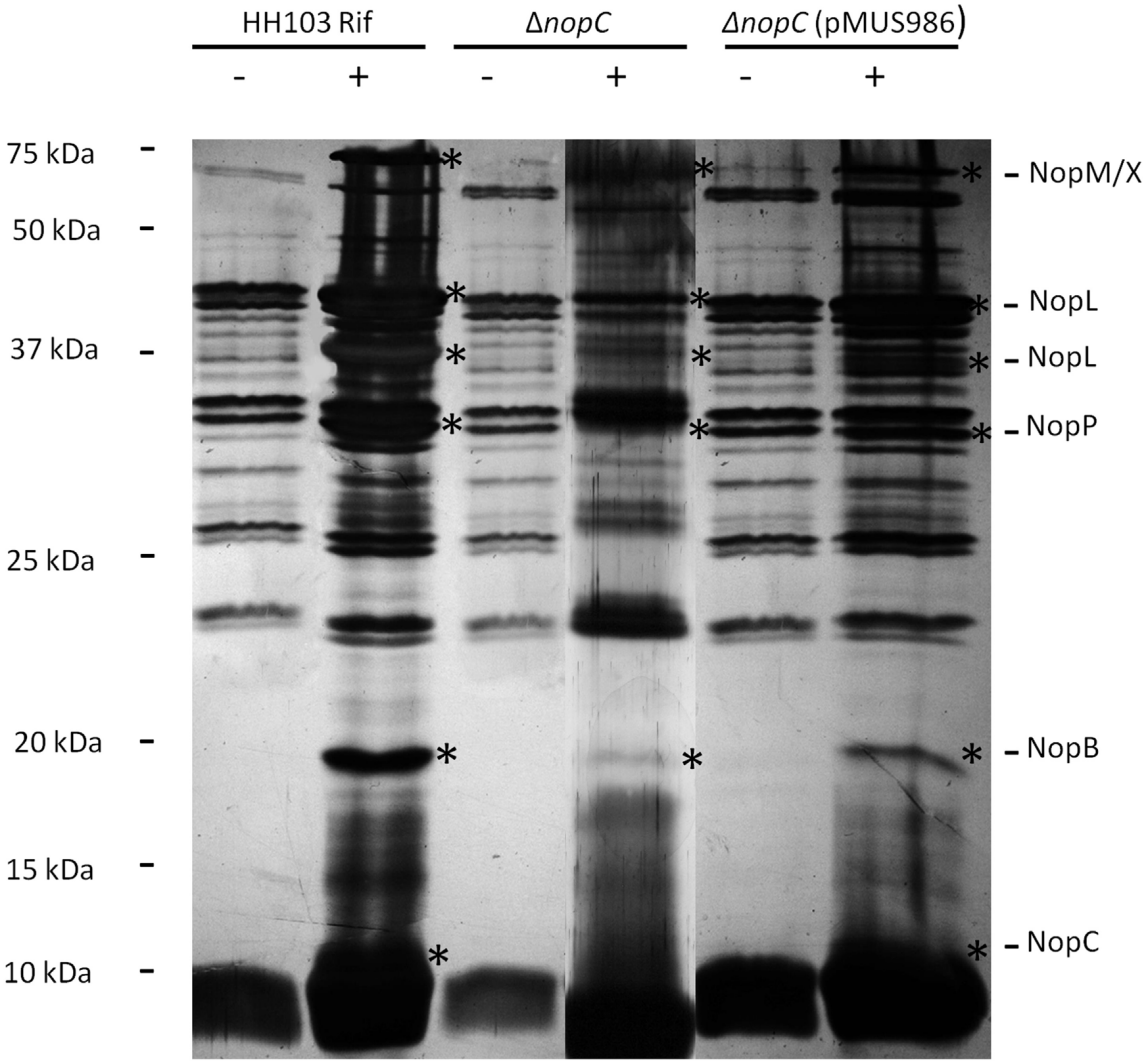
The *in frame* mutation of the *nopC* gene did not block secretion of the rest of the *S*. *fredii* HH103 Nops. Silver-stained gel of secreted extracellular proteins of HH103 Rif^R^, the HH103 Rif^R^ Δ*nopC* mutant and the *nopC* mutant complemented with plasmid pMUS986 in the absence (-) or presence (+) of genistein (3.7 μM). Proteins whose secretion depends on genistein and a functional T3SS are indicated with an asterisk and indicated on the right. Molecular masses (kDa) of the marker are shown on the left. Samples were separated by 15% SDS-PAGE.

**Fig 4:**
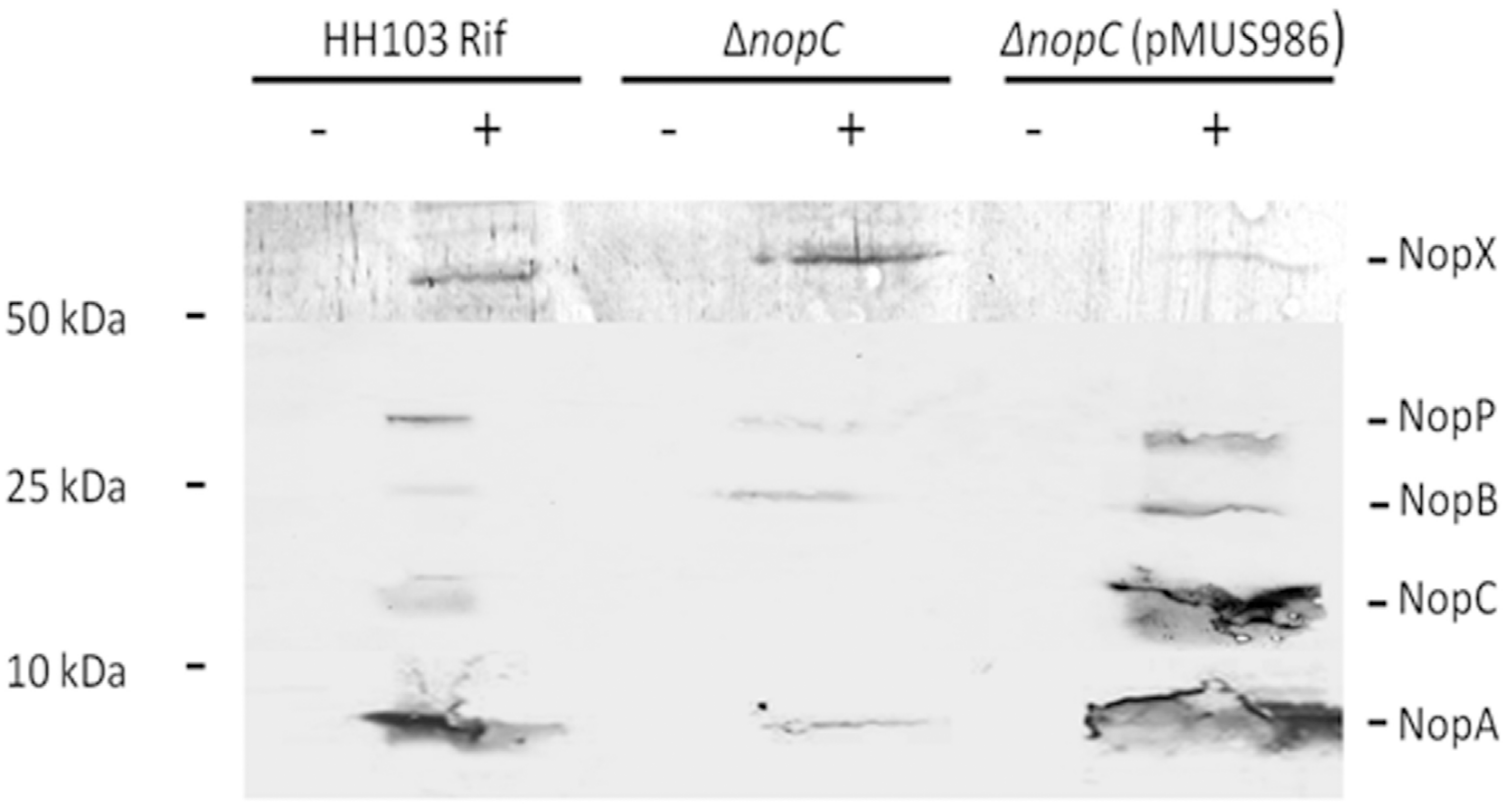
Immunodetection of several *S*. *fredii* HH103 Nops. Immunodetection of NopA, NopB, NopC, NopP, and NopX in extracellular proteins extracts of the parental strain HH103 Rif^R^, the HH103 Rif^R^ Δ*nopC* mutant and the *nopC* mutant complemented with plasmid pMUS986 in the absence (-) or presence (+) of genistein (3.7 μM). Molecular masses (kDa) of the marker are shown on the left. Samples were separated by 15% SDS-PAGE.

The analysis of protein supernatants from strain HH103 Rif^R^Δ*nopC* induced with genistein showed the presence of all the Nops secreted by HH103 with the exception of the band corresponding to NopC, indicating that type 3 secretion was not blocked in this mutant (Fig 3). This result was confirmed with western-blot analyses using antibodies raised against NopC, which showed the presence of a band of the deduced size of NopC (~ 11 kDa) in the supernatants from the parental strain HH103 Rif^R^ and the complemented strain HH103 Rif^R^Δ*nopC* (pMUS986) in the presence of genistein and not in the *nopC* mutant (Fig 4). To confirm that the absence of NopC did not affect secretion of the rest of Nops, western-blots assays with antibodies raised against NopA (~ 6 kDa), NopB (~ 20 kDa), NopP (~ 32 kDa), and NopX (~ 60 kDa) were performed. Thus, signals corresponding to the expected sizes of the Nops analyzed were detected in all the treatments when induced with genistein (Fig 4). All these results showed that the *in frame* deletion of *nopC* did not block secretion of the rest of Nops.

### The *S*. *fredii* HH103 NopC is delivered to the cytoplasm of soybean root cells

To determine whether NopC was delivered to the interior of the soybean root cells we analyzed its T3SS-dependent translocation into plant cells using the adenylate cyclase assay. The *Bordetella pertussis* calmodulin-dependent adenylate cyclase (Cya) toxin is activated within eukaryotic cells and hence increase cAMP levels. However, no activation of this protein is detected in prokaryotic cells so it can be used to identify bacterial effector proteins [38]. The Cya reporter was fused to the carboxy terminus of NopC and integrated into the symbiotic plasmid of HH103 by simple homologous recombination. This method allowed the transcription of the *nopC*-*cya* fusion by means of the native *tts* box located upstream *nopC*.

**Fig 5:**
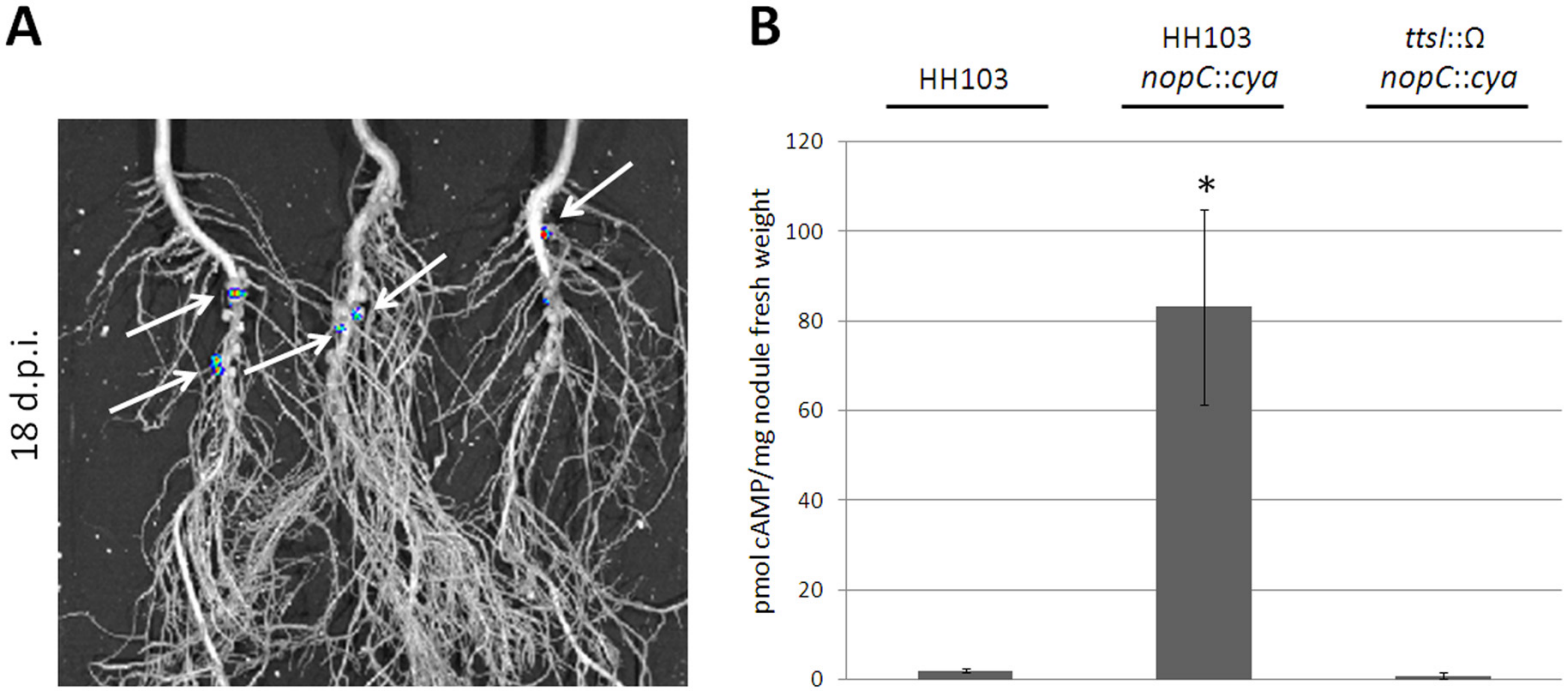
The *S*. *fredii* HH103 NopC is translocated into *Glycine max* cv. Williams 82 root cells. (A) *In vivo* monitoring of the activation of the *tts* box upstream *nopC* in vermiculite assays. Bioluminescence was measured in soybean plants inoculated with the HH103 Rif^R^ strain carrying plasmid pMUS1207 (plasmid pMP92 containing the *tts box* fused to *luxCDABE*). Bioluminiscence is shown by colored areas and indicated with arrows. (B) cAMP levels measured in soybean nodules harvested 18 d.p.i. from plants inoculated with several strains carrying the *nopC*-*cya* fusion. Data shown are the mean (± standard deviation of the mean) for two biological replicates. Each cAMP value was individually compared to that obtained in plants inoculated with the HH103 Rif^R^ strain using the Mann-Whitney non-parametrical test. Asterisks (*) indicate that numbers are significantly different at the level α = 5% (*p*< 0.05).

Previous works [24,39] demonstrated translocation of effector-Cya fusions into nodules of *Macroptilium atropurpureum* and *V*. *unguiculata*. In the case of soybean, no reports about translocation of effectors into soybean nodule cells have been published so far. In addition, some authors [40] showed that Nops can be detected in soybean infection threads but not in mature nodules. The Cya assay to determine protein translocation to the interior of root cells is time-consuming and expensive. Therefore, it was necessary to optimize the assay using only those parts of the root in which the T3SS was expressed. Thus, a previous *in vivo* monitoring of the activation of the *tts box* upstream *nopC* during symbiosis with soybean was performed using a bioluminescent reporter system. The use of bioluminescence would indicate instant expression of the *tts* genes. First, a root-flavonoid diffusion assay was used to validate the *tts* box-dependent bioluminescence. As shown in S2 Fig, a high bioluminescent signal was mainly observed in plants inoculated with the parental strain carrying plasmid pMUS1207. Then, a time-course bioluminescence monitoring assay carried out in pouches to monitor gene expression in intact plants showed that the bioluminescence was first detected in the upper region of the main root and then concentrated in the nodule, reaching a peak of expression at 21–23 days after inoculation and then declined (S3 Fig). Finally, some but not all nodules from plants grown in vermiculite, harvested at 18 d.p.i. and inoculated with the parental strain carrying the plasmid with the *tts* box-*luxCDABE* fusion, showed high bioluminescence, confirming that HH103 T3SS-regulated genes are expressed in this symbiotic organ at that time-point (Fig 5A). As expected, no bioluminescence was detected in plants inoculated with the strain carrying the empty vector (data not shown). This result allowed us the selection of young soybean nodules (18 d.p.i. nodules) as the optimum tissue for cAMP quantification. Further Cya assays showed very low levels of cAMP in the nodules formed by the parental strain and the *ttsI* mutant strain expressing the *nopC*-*cya* fusion. By contrast, cAMP accumulation was significantly higher in nodules from plants inoculated with the parental strain expressing *nopC*-*cya* (Fig 5B). These results indicated that NopC was translocated into the host cells via the T3SS of *S*. *fredii* HH103.

### NopC is beneficial for the symbiosis with *G*. *max* and *V*. *unguiculata*

To elucidate the role of NopC in the symbiosis established between HH103 and soybean, the symbiotic properties of the HH103 Rif^R^, HH103 Rif^R^Δ*nopC*, the HH103 Rif^R^Δ*nopC* mutant complemented in *cis* and the HH103 Rif^R^ *nopA*::*lacZ*-Gm^R^ strains were determined in plant infection tests (Table 3). The number and fresh mass of the nodules formed and the plant-top dry mass of the soybean plants were significantly lower (α = 5%) in plants inoculated with the *nopA* mutant strain (around 40% less in all symbiotic parameters) when compared with those inoculated with the parental strain. Interestingly, in the case of the *nopC* mutant no differences in plant-top dry masses were observed, but the fresh mass and the number of nodules formed were statistically lower (α = 5% and α = 10%, respectively) in plants inoculated with this mutant in comparison to plants inoculated with the parental strain. Complementation in *cis* of the *nopC* mutant restored all symbiotic phenotypes. These results indicated that NopC exerted a positive effect in the symbiosis between HH103 and soybean cv. Williams 82.

**Table 3:** Plant responses to inoculation of *Glycine max* cv. Williams 82 with different *Sinorhizobium fredii* HH103 strains.

Inoculant	Number of nodules	Nodules fresh mass (g)	Plant-top dry mass (g)
None	0	0	0.85 ± 0.31
HH103 Rif^R^	162.8 ± 36	2.193 ± 0.408	4.78 ± 0.67
HH103 Rif^R^ Δ*nopC*	118.2 ± 35.2**	1.436 ± 0.373*	4.2 ± 1.68
HH103 Rif^R^ Δ*nopC* complemented in *cis*	166.6 ± 51.2	2 ± 0.316	4.54 ± 0.62
HH103 Rif^R^ *nopA*::*lacZ*-Gm^R^	103.3 ± 17.3*	1.392 ± 0.393*	2.7 ± 0.67*

Data represent averages of 6 jars that contained two soybean plants. Determinations were made 6 weeks after inoculation. For each legume tested, bacteria isolated from 20 nodules formed by each inoculant showed the expected resistance markers.

*S*. *fredii* HH103 mutants were individually compared with the parental strain HH103 Rif^R^ by using the Mann-Whitney non-parametrical test. Numbers on the same column followed by an asterisk (*) are significantly different at the level α = 5%. Numbers on the same column followed by two asterisks (**) are significantly different at the level α = 10%.

Results obtained in nodulation assays with *V*. *unguiculata* showed also a positive effect of the presence of NopC in the formation of nodules, since a lower number and fresh mass of nodules were obtained in plants inoculated with the *nopC* mutant strain with respect to plants inoculated with the parental strain (differences were statistically significant in the case of the number of nodules, α = 5%) (Table 4). Interestingly, the opposite phenotype was observed in plants inoculated with the *nopA* mutant, i.e. higher number and fresh mass of nodules (α = 5%) in comparison to plants inoculated with the parental strain (Table 4). Complementation of the *nopC* mutant restored all symbiotic phenotypes.

**Table 4:** Plant responses to inoculation of *Vigna unguiculata* with different *Sinorhizobium fredii* HH103 strains.

Inoculant	Number of nodules	Nodules fresh mass (g)	Plant-top dry mass (g)
None	0	0	0.31 ± 0.14
HH103 Rif^R^	42 ± 3.2	0.517 ± 0.129	0.97 ± 0.19
HH103 Rif^R^ Δ*nopC*	31.6 ± 7.7*	0.331 ± 0.270	1.35 ± 0.38
HH103 Rif^R^ Δ*nopC* complemented in *cis*	52.4 ± 9.5	0.717 ± 0.271	1.21 ± 0.23
HH103 Rif^R^ *nopA*::*lacZ*-Gm^R^	72.2 ± 20*	0.775 ± 0.14*	0.82 ± 0.38

Data represent averages of 6 jars. Each jar contained two *V*. *unguiculata* plants. Determinations were made 6 weeks after inoculation. For each legume tested, bacteria isolated from 20 nodules formed by each inoculant showed the expected resistance markers.

*S*. *fredii* HH103 mutants were individually compared with the parental strain HH103 Rif^R^ by using the Mann-Whitney non-parametrical test. Numbers on the same column followed by an asterisk (*) are significantly different at the level α = 5%.

## Discussion

*In silico* analysis of the recently sequenced HH103 genome showed that the gene organization of the *tts* region in this strain was similar to that of *S*. *fredii* NGR234 [41]. Previous reports [21] have shown that the inactivation of the NGR234 *nopA* or *nopB* genes completely abolishes secretion of Nops and the effect of their mutation on nodulation was similar to that observed when mutating any structural component of the T3SS, suggesting that NopA and NopB were essential components of the T3SS machinery. Further experiments confirmed that both proteins were the main components of the T3SS extracellular appendages or T3SS-*pilus* [42]. In addition, bioinformatics analysis of the NopA amino acid sequence showed that this protein possesses the requisite secondary structural characteristics typical of T3SS *pili* (very high α-helical content, especially at the C-terminus region). In spite of the absence of any scientific report, NopC has also been considered in the literature as a component of the T3SS extracellular appendages. However, the analysis of the NopC amino acid sequence has revealed that this protein does not possess the secondary structures found in NopA [21]. The *nopC* gene has been found and annotated only in a very small group of rizobial genomes and similar proteins have not been detected in other pathogenic bacteria (Fig 1). Therefore, NopC can be considered a *Rhizobium*-specific T3SS secreted protein.

Results shown in this work indicated that the biosynthesis of the HH103 NopC is regulated by flavonoids and by the transcriptional regulators NodD1 and TtsI, because both gene expression and protein secretion were detected in HH103 Rif^R^ bacterial cultures supplemented with genistein but not in *nodD1* and *ttsI* mutant backgrounds (Fig 2). This regulation cascade for components or proteins secreted through the T3SS is shared by all strains possessing a T3SS, with the exception of *B*. *elkanii* USDA61 [43]. Interestingly, the inactivation of the *nopC* gene did not block secretion of the rest of Nops, indicating that NopC was not an essential component of the T3SS machinery (Figs 3 and 4). The *in frame* mutation of *nopC* did not affect transcription of *nopA*, discarding a possible polar effect of the mutation on the transcription of downstream genes (S1 Fig).

Transcription of the T3SS-genes are down-regulated in soybean bacteroids [44] and previous reports detected *S*. *fredii* USDA257 NopX (previously called NolX) in infection threads but not in mature nodules of soybean [40], suggesting that the T3SS was induced early in the symbiosis with soybean but repressed at later stages. However, results shown in this work of the *in vivo* monitoring of the activity of the *tts* box upstream *nopC* fused to *lux* genes showed that the HH103 T3SS was expressed in some but not all young soybean nodules (Fig 5A). The fact that bioluminescence was not detected in all nodules could have some explanations. First, plasmid pMUS1207 (derived from the high-copy plasmid pMP92) could be lost by part of the rhizobial population. Both bioluminescence and nitrogen fixation require high energy consumption and the promoter cloned shows very high activity with inducer flavonoids. Therefore, the presence of the plasmid and the production of bioluminescence since the early stages of the infection process could have a negative impact on bacterial fitness. In addition, luciferase needs oxygen and ATP for its activity and the atmosphere in the symbiosome, necessary for nitrogen fixation, is deficient in oxygen. Therefore, bioluminescence would not be detected in mature nodules, making this method only suitable for instant detection of gene expression in the early stages of the symbiosis, time period that coincides with the moment in which the T3SS shows its highest activity. Nevertheless, the objective of the use of this reporter was the selection of a particular root region/tissue with the best characteristics to further measure the production of cAMP in the Cya assay.

Translocation of the effectors NopE1 and NopE2 of *B*. *japonicum* USDA110 and NopP of *S*. *fredii* USDA257 within eukaryotic host cells has been determined in the symbioses with *M*. *atropurpureum* and *V*.*unguiculata*, respectively [24,39]. In both cases, effectors were fused to a *cya* reporter gene to test translocation into the plant cell cytoplasm. This specific bacterial enzyme catalyzes the cAMP production in the presence of ATP and host calmodulin-like proteins. Therefore, an increase in the cAMP levels indicates effector translocation into eukaryotic cells. Translocation of NopC to the interior of soybean cells was confirmed using this reporter assay (Fig 5B) and the quantified levels of the cAMP induced by the NopC-Cya fusion protein in soybean root cells were similar to those obtained in previous reports [24,39].

Rhizobial proteins secreted through the T3SS are involved in host-range determination and symbiotic efficiency [13]. The positive, negative or neutral effects on symbiosis of each effector are determined by the host plant. For instance, in the symbioses between *S*. *fredii* HH103 and its host legumes, the inactivation of the T3SS is beneficial for the symbioses with several cultivars of *G*. *max* (including Williams 82) and *Glycyrrhiza uralensis* and detrimental on *Erythrina variegata* [17]. Results shown in this work indicated that NopC had a positive role in the symbiosis between HH103 and both soybean cv. Williams 82 and *V*. *unguiculata*, since the mutation of this gene caused a decrease in the number and the fresh mass of the nodules formed in both plants (Tables 3 and 4). This positive effect on soybean symbiosis was also observed in plants inoculated with the *nopA* mutant strain (Table 3). Interestingly, the symbiotic effect in *V*. *unguiculata* of the mutation of *nopA* was opposite to that observed with the *nopC* mutant (Table 4), indicating that both Nops are playing different roles in the symbiotic process. Besides, results suggest that NopC is contributing, together with still unknown effectors, to an effective symbiosis with both plants.

As previously mentioned, NopC has no homologues in pathogenic bacteria and it does not possess any domain or conserved feature that could give clues about its role in symbiosis. It could be possible that NopC functions as a chaperone (T3SC) to facilitate the assembly of the secretion apparatus or secretion of effectors to the interior of the host cell. T3SCs are typically small and acidic cytoplasmic proteins that remain within the bacterial cell and some of them are encoded by a gene within an operon that carries genes encoding components of the secretion apparatus. In addition, T3SCs that bind effectors exhibit a contiguous conserved set of structural folds (α-β-β-β-α-β-β-α) and their absence results in lack of secretion of their corresponding effectors [45]. However, NopC was translocated to the interior of soybean cells, secretion of other Nops was not abolished when *nopC* was inactivated and the analysis of the NopC secondary structure showed low α-helical content at its carboxy terminus and the conserved structure of T3SCs was not detected. Further studies are necessary to determine the specific function of this *Rhizobium*-specific effector protein within the plant cell.

## Supporting Information

S1 Fig The *in frame* mutation of the *nopC* gene did not block transcription of *nopA*. *q*RT-PCR analysis of the expression of *nopA* (**A**) and *nodA* (**B**) in the parental strain HH103 Rif^R^ and the mutant strains HH103 Rif^R^ *ttsI*::Ω and HH103 Rif^R^ Δ*nopC* in the absence (-) or presence (+) of the inducer flavonoid genistein (3.7 μM). Final expression was calculated relative to the expression in the HH103 Rif^R^ strain in the absence of flavonoids. Expression data shown are the mean (± standard deviation of the mean) for three biological replicates performed at least in triplicates. Each expression value was individually compared with the HH103 Rif^R^ strain in the absence of flavonoids using the Mann-Whitney non-parametrical test. Asterisks indicate that numbers are significantly different at the level α = 5% (*p*< 0.05).
(TIF)

S2 Fig Validation of the *tts* box-*luxCDABE* fusions by *in vivo* activation with flavonoids exuded by soybean roots. *S*. *fredii* strains carrying plasmids pMUS1199 (= pMP92-*luxCDABE*) or pMUS1207 (= pMP92-*tts box*::*luxCDABE*) were assayed in squared Petri dishes with pre-germinated soybean seeds. **A**. HH103 Rif^R^ (pMUS1199). **B**. HH103 Rif^R^ (pMUS1207). **C**. HH103 Rif^R^ *nodD1*::*lacZ*-Gm^R^ (pMUS1207). **D**. HH103 Rif^R^ *ttsI*::Ω (pMUS1207). Bioluminescence was measured 72 hours after inoculation.
(TIF)

S3 Fig *In vivo* monitoring of the activation of the *tts* box upstream *nopC* using soybean plants grown in pouches. Bioluminescence was measured in soybean plants inoculated with the HH103 Rif^R^ strain carrying plasmid pMUS1207 (plasmid pMP92 containing the *tts box* fused to *luxCDABE*). Bioluminescence in nodules is indicated with a white arrow. The grey arrow shows a nodule without bioluminescence. Bioluminescence was measured at 14, 16, 19, 21, 23, 26, and 28 days after inoculation.
(TIF)
