# Supplementary figures and images for: NopC Is a Rhizobium-Specific Type 3 Secretion System Effector Secreted by Sinorhizobium (Ensifer) fredii HH103
Source: PLoS One. 2015 Nov 16;10(11):e0142866. doi: 10.1371/journal.pone.0142866 (PMC4646503; doi:10.1371/journal.pone.0142866)

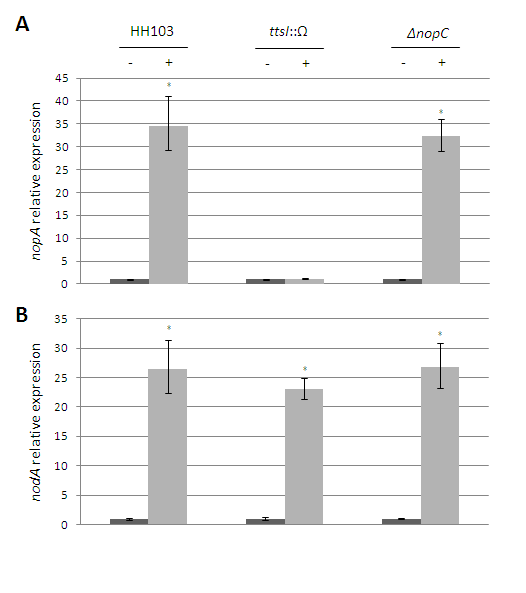

Supplement: S1 Fig — qRT-PCR analysis of the expression of nopA (A) and nodA (B) in the parental strain HH103 RifR and the mutant strains HH103 RifR ttsI::Ω and HH103 RifR ΔnopC in the absence (-) or presence (+) of the inducer flavonoid genistein (3.7 μM). Final expression was calculated relative to the expression in the HH103 RifR strain in the absence of flavonoids. Expression data shown are the mean (± standard deviation of the mean) for three biological replicates performed at least in triplicates. Each expression value was individually compared with the HH103 RifR strain in the absence of flavonoids using the Mann-Whitney non-parametrical test. Asterisks indicate that numbers are significantly different at the level α = 5% (p< 0.05). (TIF) [file pone.0142866.s001.TIF]

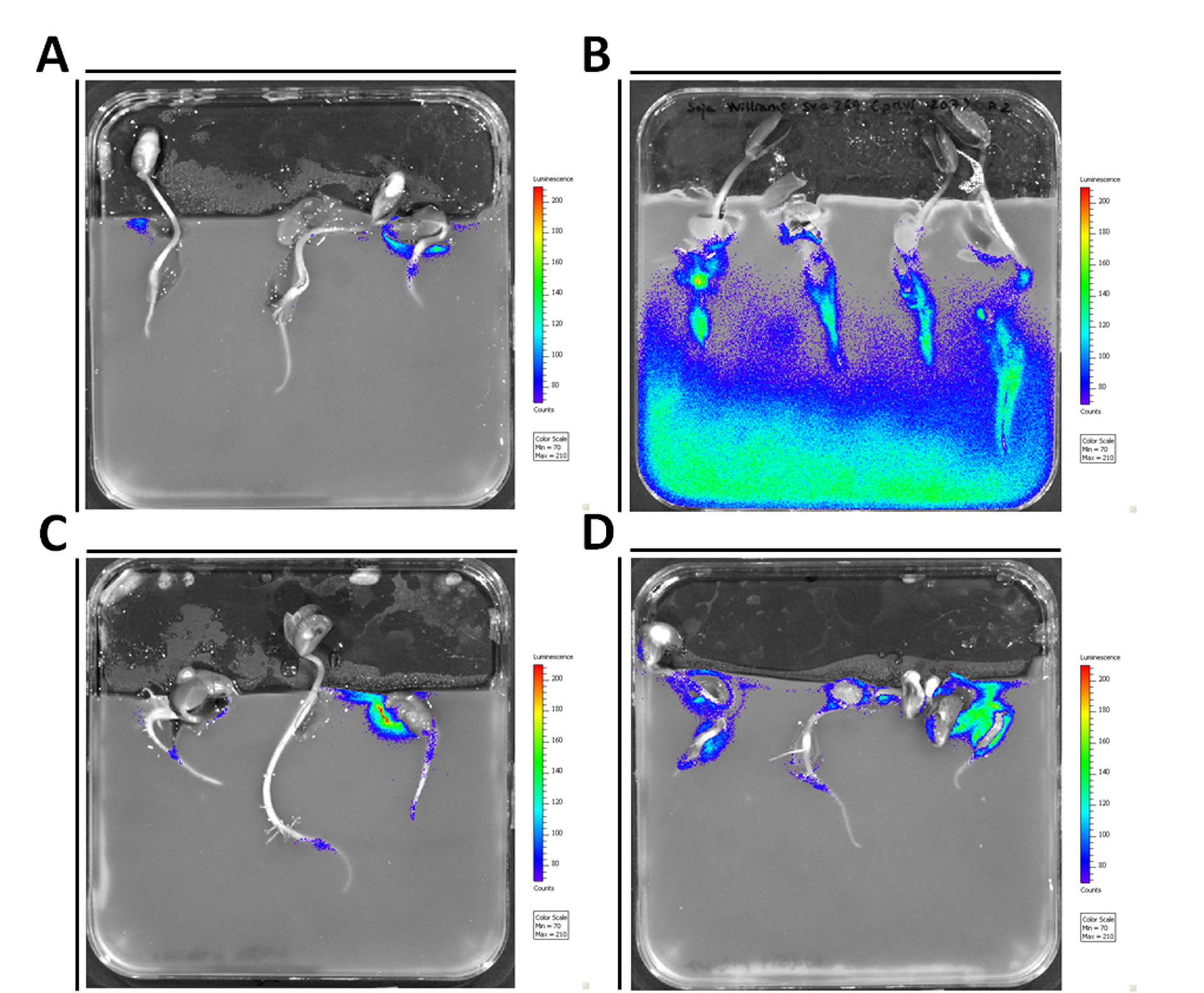

Supplement: S2 Fig — S. fredii strains carrying plasmids pMUS1199 (= pMP92-luxCDABE) or pMUS1207 (= pMP92-tts box::luxCDABE) were assayed in squared Petri dishes with pre-germinated soybean seeds. A. HH103 RifR (pMUS1199). B. HH103 RifR (pMUS1207). C. HH103 RifR nodD1::lacZ-GmR (pMUS1207). D. HH103 RifR ttsI::Ω (pMUS1207). Bioluminescence was measured 72 hours after inoculation. (TIF) [file pone.0142866.s002.tif]

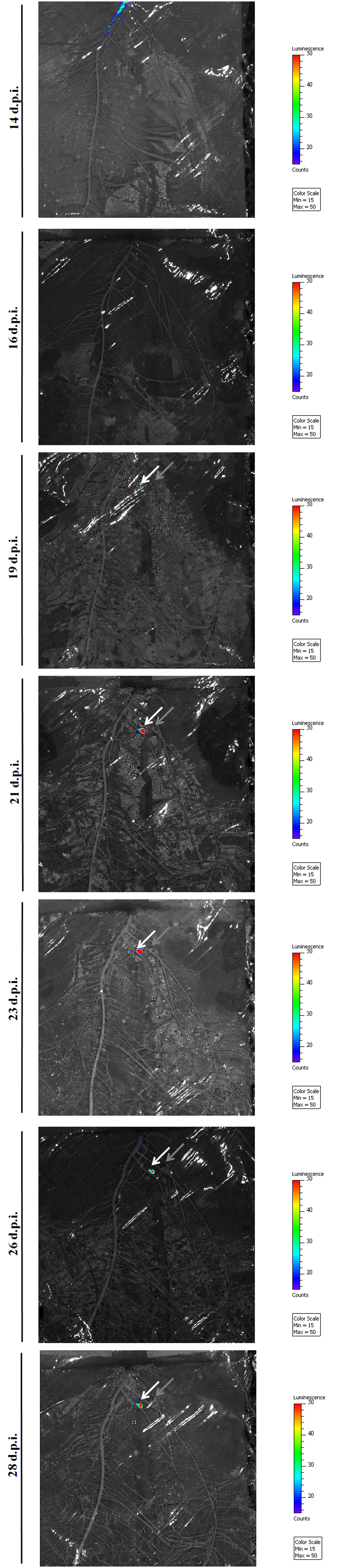

Supplement: S3 Fig — Bioluminescence was measured in soybean plants inoculated with the HH103 RifR strain carrying plasmid pMUS1207 (plasmid pMP92 containing the tts box fused to luxCDABE). Bioluminescence in nodules is indicated with a white arrow. The grey arrow shows a nodule without bioluminescence. Bioluminescence was measured at 14, 16, 19, 21, 23, 26, and 28 days after inoculation. (TIF) [file pone.0142866.s003.tif]
